# Supplementary material for: Effects of moderate physical activity on diabetic adhesive capsulitis: a randomized clinical trial
Source: PeerJ. 2024 Sep 18;12:e18030. doi: 10.7717/peerj.18030 (PMC11416079; doi:10.7717/peerj.18030)
Supplement: Supplemental Information 3 [file peerj-12-18030-s003.pdf]

EFFECTS OF MODERATE PHYSICAL ACTIVITY ON DIABETIC ADHESIVE CAPSULITIS: A RANDOMIZED CLINICAL TRIAL

SYNOPSIS

AMNA TOSEEF
Registration Number: 28710

Master of Science in Physical Therapy
(Sports Physical Therapy)

Riphah College of Rehabilitation & Allied Health Sciences

RIPHAH INTERNATIONAL UNIVERSITY
ISLAMABAD
2021

AMNA TOSEEF
Registration Number: 28710

PROF. WAQAAR AHMED AWAAN
Supervisor

In Partial Fulfilment of Requirements For the Award of Degree of Master in Science of Physical Therapy (Sports Physical Therapy)

Riphah College of Rehabilitation & Allied Health Sciences
RIPHAH INTERNATIONAL UNIVERSITY
ISLAMABAD
2021

RIPHAH INTERNATIONAL UNIVERSITY
ACADEMIC PROGRESS REPORT

As on ______________________
For the period *from* _________________ *to* _______________

1. Personal Information of Scholar:

Name:	Amna Toseef
Registration No.:	28710
Program:	MSPT(SPT)
Faculty/Department:	College of Rehabilitation and Allied Health Sciences
Email:	amnatoseef@hotmail.com

2. Academic Progress:

Admission Date:	
Status of Coursework (Credit hours completed and remaining):	2nd Semester in progress.
Expected Date of Completion of Research Work:	1 year after the approval.
Expected Date of Completion of Program:	
Last GPA and CGPA (Please attach result of each semester):	3.68 GPA.

3. Research Topic:

Topic of Research:	Effects of Moderate Physical Activity on Diabetic Adhesive Capsulitis: A randomized clinical trial
Date of Approval	
Name of Supervisor	Dr. Waqar Ahmad Awan.
Name of Co-Supervisor (if any):	None
Status of Research Work	In progress.

4. Employment Status:

Unemployed	No
Employed (job place, title, and status—i.e., on study leave or otherwise)	Physiotherapist in Muzaffarabad COVID Isolation Hospital. MZD

Please Note: The scholars under HEC Indigenous 5000 Fellowship Program shall not undertake any employment whether paid or otherwise at any stage during their course of study of the program.

Dated: _________________________ Signature of Scholar: [signature] ________

Remarks of the Supervisor:

The timely responded the all task assigned. This study will contribute significantly to the literature
regarding the management of the diabetic adhesive capsulitis patients.

5. Overall progress: (please tick only one)

Poor	Satisfactory	Good	Very Good	Excellent
		✓		

Verified/Certified by	Countersigned by
**Supervisor**	**Associate-Dean post Graduate Program**
Name: Prof Waqar Ahmed Awan	Name: Prof Dr Imran Amjad
Signature:	Signature:
Date: December 13, 2021	Date:

SIGNATURES

Effects of Moderate Physical Activity on Diabetic Adhesive Capsulitis: A randomized clinical trial

Name of Student: Amna Toseef

Registration No: 28710

Prof Dr Waqar Ahmed Awan (Supervisor)

Dr Saeed Bin Ayaz (Co-Supervisor)

1. INTRODUCTION

Adhesive Capsulitis (AC) is a self-limiting condition also known as frozen shoulder. Mostly patients present with pain, stiffness and decreased shoulder ROM at gleno-humeral joint actively and passively(1). 2 major types; Primary or idiopathic adhesive capsulitis is characterized by a gradual onset of pain and stiffness at the gleno-humeral joint without a specific cause. Secondary adhesive capsulitis is known to be caused by several predisposing factors like trauma, shoulder surgery, diabetes mellitus and thyroid dysfunctions(2). The prevalence of AC is 2-5% in general population. Mostly women between age of 40-60 years are diagnosed with this condition. AC is an idiopathic condition with increased prevalence in patients with diabetes mellitus and hyperthyroidism(1). Patients with Diabetes Mellitus (DM) are found to be affected 5 times more with AC. Prevalence of Diabetic Adhesive Capsulitis is 13.4%. This condition usually lasts for 1-3 years without any treatment and affects patient's ADL's and IADL's (3).

HbA1c is seen to have relationship with AC. When HbA1c level was greater than 7 each unit time, then the risk of AC was raised by 2.77%. Patients with poor glycemic control over longer periods of time have increased risk(4). Presence of AC is also thought to be the indicator of developing diabetes in pre-diabetic population, for which screening should be conducted. 71.5% having AC were also diabetic(5). Proper mechanism of association is still vague. In DM, inflammatory markers are raised which cause micro vascular complications. In AC, capsular fibrosis and synovial inflammation occurs with elevated levels of pro inflammatory cytokines (interleukin-1B, IL-6 and tumor necrosis factor) in them. Hence, DM related chronic inflammatory process with increased growth factor expression, which in turn leads to joint synovitis and subsequent capsular fibrosis(6,7).

The most effective treatment for AC is uncertain. Management can be operative and non-operative. Arthroscopic release in patients showed excellent results in 90% and 10% had fair outcomes(3). Strong evidence suggests that steroid injections and laser therapy are effective in short-term only. Moderate evidence was found in favor of mobilization techniques in the short and long term(8). Exercises alone and exercises with manual therapy are equally effective in the management of AC of the shoulder joint(9).Certain physical therapy techniques and modalities are

strongly recommended for pain relief, improvement of ROM, and functional status in patients with AC, while others are either moderately or mildly recommended. However, the efficacy of one treatment modality over another is uncertain(10). Low-quality evidence suggests large effects of joint mobilization plus exercises on AC in people with diabetes (11).

Physical activity remains a key element in prevention and management of type 2 diabetes and its complications. Insulin resistance, poor glycemic control and raised A1C cause inflammation which might lead to AC development. Practice of three brisk walking sessions per week at a rate of 30 minutes each has beneficial impact on physical performance, A1C reduction, glycemic control and complication reduction in diabetic patients(12). Appropriate lifestyle interventions may delay or prevent DM in individuals with impaired glucose tolerance. Any indoor or outdoor activity 30-120 min after a meal helps reduce blood glucose. Resistance exercise increases the quantity and strength of muscles, expels fat from muscles, improves insulin resistance, enhances collagen production and gets rid of the sugar attached to collagen fibers causing restriction. Adult diabetes patients should engage in resistance exercise 2 to 3 times a week. Heavier load is more effective for improving blood glucose levels and strength, it also delays the complications such as AC and cardiovascular diseases caused by diabetes (13). Physical activity and exercise should be recommended and prescribed to all individuals with diabetes as part of management of glycemic control and overall health. Specific recommendations and precautions will vary by the type of diabetes, age, activity done and presence of diabetes-related health complications (14).

The adoption and maintenance of physical activity is critical for blood glucose management and overall health in individuals with diabetes. In this Position Statement, we provide a clinically oriented review and evidence based recommendations regarding physical activity and exercise in people with type 2 diabetes.

Since the patients with diabetes mellitus are more prone to cause musculoskeletal injuries and progression of recovery is very slow also there is limited literature proving the effects of moderate physical activity in addition to conventional physical therapy in treatment of Diabetic Adhesive Capsulitis. In this study, it is hypothesized that moderate physical activity in addition to conventional physical therapy significantly improves the symptoms of AC and progresses the rate of

recovery, so objective of the study aims to determine the effects of performing moderate physical activity in addition with standard protocol of treating diabetic adhesive capsulitis.

2. OBJECTIVE & HYPOTHESIS OF STUDY

2.1. Objectives

To determine the effects of moderate physical activity on adhesive capsulitis in patients with diabetes mellitus.

2.2. Hypothesis

2.2.1. Research Hypothesis: Effects of moderate physical activity are significant to improve adhesive capsulitis in patients with diabetes mellitus.

2.2.2. Null Hypothesis: Effects of moderate physical activity are not significant to improve adhesive capsulitis in patients with diabetes mellitus.

3. MATERIAL & METHODS

3.1. Design

The study design will be Randomized Controlled Trial (RCT) having 2 groups i.e: Experimental (Moderate Physical Activity) and Control (Conventional training)

3.2. Sample Size

Sample size is calculated by using G*Power. A total of n=44 sample size was calculated through G power, keeping effect size small (0.25), α error margin at 0.05. To avoid β error probability, the power (1- β) was set at 0.95%.

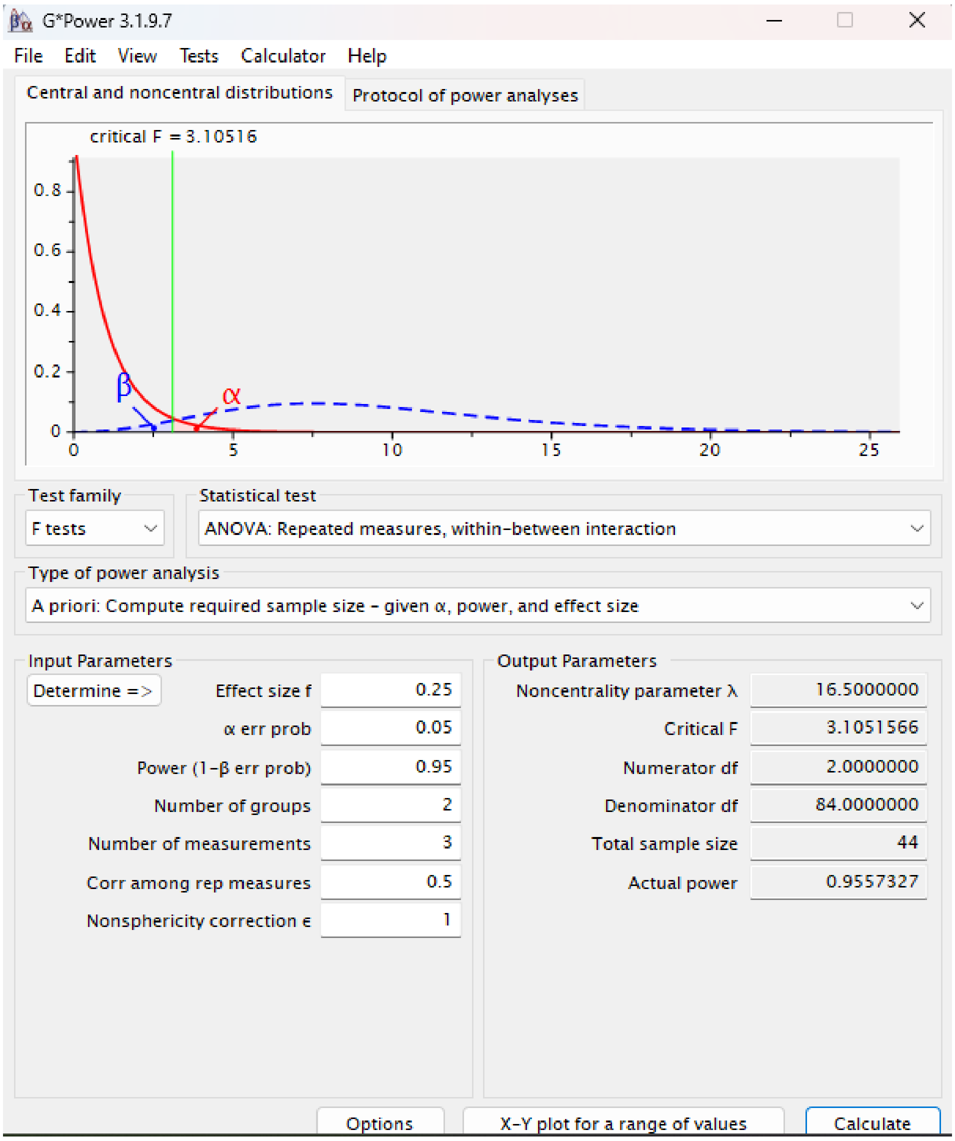

3.3. Study Duration

The study duration would be 1 year after the approval from the research board.

3.4. Sampling Technique

Non probability Convenient Sampling Technique would be used. Group randomization would be done by using sealed envelope method.

3.5. Study Setting

The study will be conducted in Rehabilitation department of SKBZ Combined Military Hospital, Muzaffarabad.

3.6. Selection Criteria

3.6.1. Inclusion Criteria

- Type II Diabetes Mellitus patients (HbA1c > 6.5%)
- Patients having stage I and II Adhesive Capsulitis with positive capsular pattern
- Age between 40 to 65 years old
- Both male and female

3.6.2. Exclusion Criteria

- Any other shoulder disorder
- Lower limb injuries
- Post-surgical and trauma patients
- Diabetic foot ulcer patients
- Mobility disorder
- Rheumatologic disorder

3.7. Tools

Following tools would be used for the assessment of diabetic frozen shoulder during the study.

- **Numeric Pain Rating Scale:** Shoulder pain was assessed using numeric pain rating scale (NPRS), a reliable (Cronbach's α=0.94) and valid tool (CI = 0.96 to 0.98) for assessing pain (22)
- **Goniometer:** It is a tool used to measure the angle of joint or joint range of motion. It has shown reliability of (ICC=0.94) and validity (ICC=0.94) (17).
- **HbA1c test:** The HbA1c test, also known as the haemoglobinA1cis average blood glucose level for past two to three months. The normal range for the

hemoglobin A1c level is between 4% and 5.6%. Between 5.7% and 6.4%indicates pre-diabetes. 6.5% or higher indicates diabetes. Validity (r=0.96 and 0.99) and reliability (r=0.95 and 0.97) (18).

- **DASH Questionnaire:** The DASH (Disabilities of the Arm, Shoulder and Hand) Outcome Measure is a 30-item, self-reported questionnaire that measures physical function and symptoms in musculoskeletal disorders of the upper limb. Validity and Reliability of this scale is (ICC=0.95) and (ICC=0.92) respectively (19).

3.8 Intervention:

All patients will receive a total of 30 sessions, 5 sessions per week for 6 weeks. The duration of each session in CPT group (Control) will be 30 minutes, while in moderate physical activity (MPA) group (Experimental) 60 minutes. Before intervention, a brief introduction of interventions will be given to both groups. The experimental group will be further instructed to perform physical activity on treadmill, walking speed and overall handling of the machine. The Physical therapist will supervise the physical activity on the treadmill. Initially, walking will be started with patient comfortable speed and then increased to the desired level. (Table 1)

Table 1: Intervention Protocol

<table>
<tr><th></th><th>Experimental</th><th>Control</th></tr>
<tr><td>Conventional Physical Therapy</td><td colspan="2">Hot pack and TENS for 10 minutes at the affected shoulder.
Passive shoulder mobilizations will be performed initially at pain free range in anterior, posterior, and inferior direction (10 reps x 1 set).
Shoulder rolls, pendulum stretch, cross body arm stretch and towel stretch (10 reps x 1 set) will be actively performed by the patient with-in limits of pain (20).</td></tr>
<tr><td>Moderate Physical Activity</td><td>Moderate physical activity on treadmill, brisk walk will be performed 5 days a week for 30 minutes at 4 mph speed (3-6 METs) with warm up for a 5 minutes at low speed and then at the end speed will also decrease for a 5 minutes (21).</td><td>-</td></tr>
</table>

3.9 Data Collection Procedure

The data collection procedure will commence following the approval from the Research Ethical Review Committee of Riphah International University and SKBZ Combined Military Hospital. The study is designed to prioritize participant safety, ensuring no potential physical or emotional harm to any individual involved. To protect the privacy of participants, all collected data will be coded and kept strictly confidential, with no disclosure to any unauthorized parties.

Informed consent will be obtained from each participant, and their signatures on the consent form will affirm their voluntary participation in the research project. The researcher will thoroughly explain the detailed procedure, including the associated risks and benefits, to the patients prior to their involvement.

Both study groups will be monitored using a standardized protocol throughout the research duration. The data for outcome variables will be measured at baseline, third week, and sixth week, except for HBA1c, which will only be measured at baseline and after the sixth week.

3.10 Data Analysis

The study will utilize statistical analyses to assess the interaction effects between interventions and assessment levels, employing mixed ANOVA. For main effects, repeated measure ANOVA will be applied on variables such as VAS (Visual Analog Scale), shoulder range of motion (ROMs), and DASH (Disabilities of the Arm, Shoulder, and Hand) score, with pairwise comparisons. Regarding the assessment of HBA1c at baseline and after the 6th week, within-group changes will be evaluated using the paired sample t-test. To compare the outcomes between the study groups, independent t-tests will be employed. These rigorous statistical methods will help us gain valuable insights into the effectiveness of the interventions and their impact on the measured variables.

3.11 Gant Chart

	Nov-2021	Jan-2022	Feb-2022	Sep-2022	Nov-2022	Dec-2022	Jan-2023
**Title approval**	✓						
**Synopsis Defence**		✓					
**BASR Approval**		✓					
**Data collection**			✓				
**Article writing**				✓			
**Report writing**					✓		
**Final presentation**						✓	
**Report submission**							✓

REFERENCES

1. Ramirez J. Adhesive capsulitis: diagnosis and management. American family physician. 2019 Mar 1;99(5):297-300.
2. Le HV, Lee SJ, Nazarian A, Rodriguez EK. Adhesive capsulitis of the shoulder: review of pathophysiology and current clinical treatments. Shoulder & elbow. 2017 Apr;9(2):75-84.
3. Zreik NH, Malik RA, Charalambous CP. Adhesive capsulitis of the shoulder and diabetes: a meta-analysis of prevalence. Muscles, ligaments and tendons journal. 2016 Jan;6(1):26.
4. Chan JH, Ho BS, Alvi HM, Saltzman MD, Marra G. The relationship between the incidence of adhesive capsulitis and hemoglobin A1c. Journal of shoulder and elbow surgery. 2017 Oct 1;26(10):1834-7.
5. Rai SK, Kashid M, Chakrabarty B, Upreti V, Shaki O. Is it necessary to screen patient with adhesive capsulitis of shoulder for diabetes mellitus?.Journal of family medicine and primary care. 2019 Sep;8(9):2927.
6. Huang YP, Fann CY, Chiu YH, Yen MF, Chen LS, Chen HH, Pan SL. Association of diabetes mellitus with the risk of developing adhesive capsulitis of the shoulder: a longitudinal population-based followup study. Arthritis care & research. 2013 Jul;65(7):1197-202.
7. Kabbabe B, Ramkumar S, Richardson M. Cytogenetic analysis of the pathology of frozen shoulder. International journal of shoulder surgery. 2010 Jul;4(3):75.
8. Favejee MM, Huisstede BM, Koes BW. Frozen shoulder: the effectiveness of conservative and surgical interventions—systematic review. British journal of sports medicine. 2011 Jan 1;45(1):49-56.
9. Ali SA, Khan M. Comparison for efficacy of general exercises with and without mobilization therapy for the management of adhesive capsulitis of shoulder-An interventional study. Pakistan journal of medical sciences. 2015 Nov;31(6):1372.
10. Nakandala P, Nanayakkara I, Wadugodapitiya S, Gawarammana I. The efficacy of physiotherapy interventions in the treatment of adhesive capsulitis: A systematic review. Journal of Back and Musculoskeletal Rehabilitation. 2021 Jan 1;34(2):195-205.
11. Sana'a AA, Nazari G, Bobos P, MacDermid JC, Overend TJ, Faber K. Effectiveness of nonsurgical interventions for managing adhesive capsulitis in patients with diabetes: a systematic review. Archives of physical medicine and rehabilitation. 2019 Feb 1;100(2):350-65.
12. LAKHDAR D, Mustapha DI, KHALED MB. Effectiveness of Brisk Walking Exercise on Glycaemic Control and Cardio-Vascular Risk Factors in Patients with Type 2 Diabetes.Journal of Drug Delivery and Therapeutics. 2020 Aug 15;10(4-s):53-62.
13. Wang B, Mu XL, Zhao J, Jiang HP, Li SS, Yan G, Hua YY, Ren XY, Xing LX, Liang Y, Zhang SD. Effects of lifestyle interventions on rural patients with type 2 diabetes mellitus. World journal of diabetes. 2020 Jun 15;11(6):261.

14. Bouça-Machado R, Rosário A, Caldeira D, Castro Caldas A, Guerreiro D, Venturelli M, et al. Physical activity, exercise, and physiotherapy in parkinson's disease: defining the concepts. Movement disorders clinical practice. 2020;7(1):7-15.
15. Juel NG, Brox JI, Brunborg C, Holte KB, Berg TJ. Very high prevalence of frozen shoulder in patients with type 1 diabetes of≥ 45 years' duration: The Dialong Shoulder Study. Archives of physical medicine and rehabilitation. 2017 Aug 1;98(8):1551-9.
16. Bijur PE, Silver W, Gallagher EJ. Reliability of the visual analog scale for measurement of acute pain.Academic emergency medicine. 2001 Dec;8(12):1153-7.
17. Kolber MJ, Hanney WJ. The reliability and concurrent validity of shoulder mobility measurements using a digital inclinometer and goniometer: a technical report. International journal of sports physical therapy. 2012 Jun;7(3):306.
18. Carter JS, Houston CA, Gilliland SS, Perez GE, Owen CL, Pathak DR, Little RR.Rapid HbA1c testing in a community setting.Diabetes Care. 1996 Jul 1;19(7):764-7.
19. Kitis A, Celik E, Aslan UB, Zencir M. DASH questionnaire for the analysis of musculoskeletal symptoms in industry workers: a validity and reliability study. Applied ergonomics. 2009 Mar 1;40(2):251-5.
20. Järvinen TA, Järvinen TL, Kääriäinen M, Kalimo H, Järvinen M. Muscle injuries: biology and treatment. The American journal of sports medicine. 2005;33(5):745-64
21. Umpierre D, Ribeiro PA, Kramer CK, LeitŃo CB, Zucatti AT, Azevedo MJ, et al. Physical activity advice only or structured exercise training and association with HbA1c levels in type 2 diabetes: a systematic review and meta-analysis. JAMA. 2011;305(17):1790-99
22. Modarresi S, Lukacs MJ, Ghodrati M, Salim S, MacDermid JC, Walton DM. A Systematic Review and Synthesis of Psychometric Properties of the Numeric Pain Rating Scale and the Visual Analog Scale for Use in People With Neck Pain. The Clinical Journal of Pain. 2022;38(2):132-48. doi: 10.1097/ajp.0000000000000999. PubMed PMID: 00002508-202202000-00008.

ANNEXURES

Annexure 1: Consent form (English)

Consent Form for Participation in a Research Study

Riphah College of Rehabilitation and Allied Health Sciences, Potohar Campus Rawalpindi.

This research study **''Effects of moderate physical activity on diabetic adhesive capsulitis''** is going to be held in hospital.

All data collected from you will be coded in order to protect your identity, and would not be disclosed to anyone. Following the study there will be no way to connect your name with your data. Your answers to the questions will not affect you.

If you have any questions or concerns about this study or if any problems arise, please contact Riphah College of Rehabilitation and Allied Health Sciences, Potohar Campus at 0092,. If you have any questions or concerns about your rights as a research participant, please contact the Riphah College of Rehabilitation and Allied Health Sciences Review Board at 092-512891835, ext. 1198.

Your participation in this research study is voluntary. You may choose not to participate and you may withdraw your consent to participate at any time. You will not be penalized in any way if you decide not to participate or to withdraw from this study.

In the unlikely event of injury resulting from this research, Riphah College of Rehabilitation and Allied Health Sciences, Potohar campus is not able to offer financial compensation nor to absorb the costs of medical treatment. However, assistance will be provided to research subjects in obtaining emergency treatment and professional services that are available to the community generally at nearby facilities.

My signature below acknowledges my consent to voluntarily participate in this research project. Such participation does not release the investigator(s), sponsor(s) or granting agency (ies) from their professional and ethical responsibility to me.

I have read this consent form and have been given the opportunity to ask questions. I give my consent to participate in this study.

Participant's signature________________________________ Date:_________________

Anexure 2: Consent form (Urdu)

رفاہ کالج آف ری ہیبلٹیشن اینڈ الائیڈ ہیلتھ سائنسز ، پوٹوہار کیمپس پنڈی۔

اس تحقیقی مطالعہ ". بے قابو ذیابیطس میلیتس کے مریضوں میں چپکنے والی کیپسولائٹس پر اعتدال پسند جسمانی سرگرمی کے اثرات۔ مضامین کی بھرتی کے بعد ، ہر ایک شرکا کی جائزہ درست سوالنامے کے ذریعہ کی جائے گی۔

آپ سے جمع کردہ تمام کوائف کو اپنی شناخت کے تحفظ کے لئے کوڈ کیا جائے گا ، اور کسی کو بھی انکشاف نہیں کرنا چاہئے۔ مطالعہ کے بعد آپ کے نام کو اپنے ڈیٹا سے مربوط کرنے کا کوئی طریقہ نہیں ہوگا۔ سوالوں کے جوابات آپ کو متاثر نہیں کریں گے۔

اگر آپ کو اس مطالعے کے بارے میں کوئی سوالات یا خدشات ہیں یا کوئی پریشانی پیش آتی ہے تو ، براہ کرم رفاہ کالج آف بحالی اور الائیڈ ہیلتھ سائنسز ، پوٹوہار کیمپس 0092 پر رابطہ کریں۔ اگر آپ کو بطور تحقیقی شریک اپنے حقوق کے بارے میں کوئی سوالات یا خدشات ہیں تو برائے مہربانی رفاہ کالج آف ری ہیبیٹیشن اینڈ الائیڈ ہیلتھ سائنسز ریویو بورڈ سے رابطہ کریں۔ 1198-

اس تحقیقی مطالعہ میں آپ کی شرکت رضاکارانہ ہے۔ آپ حصہ نہ لینے کا انتخاب کرسکتے ہیں اور آپ کسی بھی وقت حصہ لینے کے لئے اپنی رضامندی واپس لے سکتے ہیں۔ آپ کو کسی بھی طرح سے سزا نہیں دی جائے گی اگر آپ اس مطالعہ میں حصہ نہ لینے یا دستبردار نہ ہونے کا فیصلہ کریں۔

اس تحقیق کے نتیجے میں ہونے والے کسی چوٹ کے غیرمعمولی واقعہ میں ، پوپہر کیمپس ریپہ کالج آف ری ہیبیٹیشن اینڈ الائیڈ ہیلتھ سائنسز ، مالی معاوضے کی پیش کش نہیں کرسکتا ہے اور نہ ہی طبی علاج معالجے کے اخراجات کو جذب کرنے کے لئے۔ تاہم ، ہنگامی علاج اور پیشہ ورانہ خدمات کے حصول میں مضامین کی تحقیقات میں مدد فراہم کی جائے گی جو عام طور پر قریبی سہولیات پر معاشرے کو دستیاب ہیں۔

ذیل میں میرے دستخط اس تحقیقی منصوبے میں رضاکارانہ طور پر حصہ لینے کے لئے میری رضامندی کو تسلیم کرتے ہیں۔ اس طرح کی شرکت تفتیش کاروں ، کفیل (سپانسرز) یا گرانٹ ایجنسی (ies) کو اپنی پیشہ ورانہ اور اخلاقی ذمہ داری سے مجھ پر رہا نہیں کرتی ہے۔

میں نے رضامندی کا یہ فارم پڑھ لیا ہے اور مجھے سوالات کرنے کا موقع ملا ہے۔ میں اس مطالعہ میں حصہ لینے کے لئے اپنی رضامندی دیتا ہوں۔

شریک کے دستخط ______________________________ تاریخ: ___________________

DEMOGRAPHICS

Name: _____________ Age: _________ Gender: Male / Female

When you were first diagnosed with diabetes? ____________ year / months

Is your diabetes controlled? Yes / No

Do you use for your diabetes management? Insulin/ Medication

Which Medicine do you use? ____________

Do you have co-morbidities related to diabetes? Yes / No

If yes, please specify ____________

Do you walk or exercise? If Yes, then how often do you walk?

Once a week / Twice a week /Daily

HbA1c: __________

BMI: ___________

PAIN SYMPTOMS:

Shoulder pain: Yes/ No

If Yes, for how long ____________

Which side: Right/ Left

Which type: a) Continuous / Intermittent

b) Sharp / Dull

Shoulder Ranges:

SHOULDER RANGES	ABDUCTION (0-150 degree)	EXTERNAL ROTATION (0-90 degree)	INTERNAL ROTATION (0-90 degree)
Affected side R/ L			

Visual Analogue Scale:

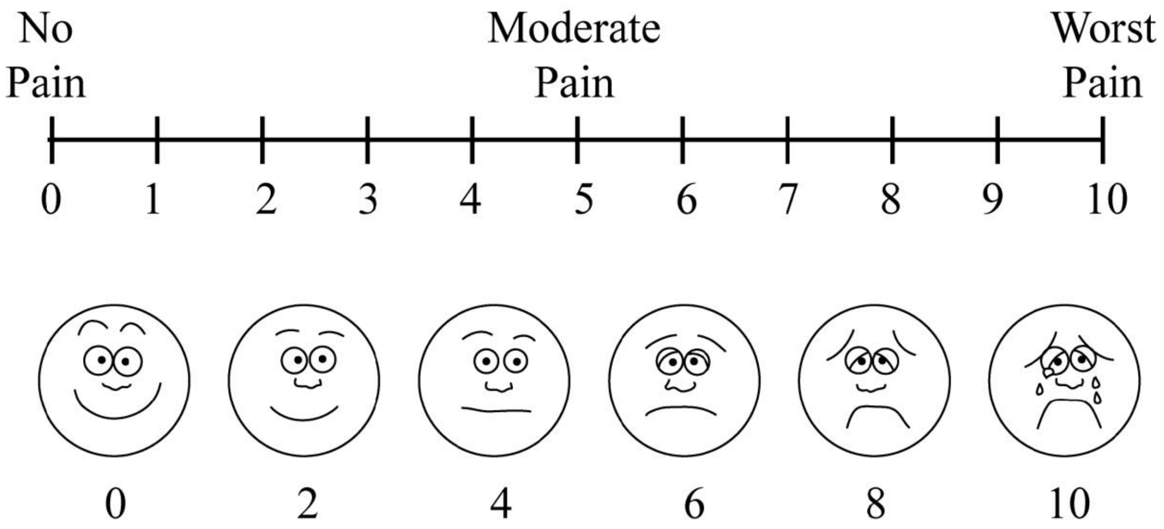

Current Score: _____	Last Score:_____
Current Pain: _____	Last Pain: _____

Disabilities of the Arm, Shoulder and Hand (DASH) Questionnaire

Patient Name:________________________ **Date:** __________ **Therapist:**________________ **Clinic:**__________________

Instructions:
This questionnaire asks about your symptoms as well as your ability to perform certain activities. Please answer every question, based on your condition within the last week by marking the appropriate answer. If you did not have the opportunity to perform an activity in the past week, please make your best guess as to which response would be most accurate. It doesn't matter which hand or arm you use to perform the activity, please answer based on your ability regardless of task.

Part A – Daily Activities
Please rate your ability to do the following activities in the last week by marking the box that corresponds to the most appropriate response.

		NO DIFFICULTY	MILD DIFFICULTY	MODERATE DIFFICULTY	SEVERE DIFFICUTLY	UNABLE
1	Open a tight or new jar	1	2	3	4	5
2	Write	1	2	3	4	5
3	Turn a key	1	2	3	4	5
4	Prepare a meal	1	2	3	4	5
5	Push open a heavy door	1	2	3	4	5
6	Place an object on a shelf above your head	1	2	3	4	5
7	Do heavy household chores (e.g., wash walls, wash floors)	1	2	3	4	5
8	Garden or do yard work	1	2	3	4	5
9	Make a bed	1	2	3	4	5
10	Carry a shopping bag or briefcase	1	2	3	4	5
11	Carry a heavy object (over 10 lbs.)	1	2	3	4	5
12	Change a light bulb overhead	1	2	3	4	5
13	Wash or blow dry your hair	1	2	3	4	5
14	Wash your back	1	2	3	4	5
15	Put on a pullover sweater	1	2	3	4	5
16	Use a knife to cut food	1	2	3	4	5
17	Recreational activities which require little effort (e.g., card playing, knitting, etc)	1	2	3	4	5
18	Recreational activities, in which you take some force or impact through your arm, shoulder or hand (e.g., golf, hammering, tennis, etc.)	1	2	3	4	5
19	Recreational activities in which you move your arm freely (e.g., playing frisbee, badminton, etc.)	1	2	3	4	5
20	Manage transportation needs (getting from one place to another)	1	2	3	4	5
21	Sexual activities	1	2	3	4	5

Disabilities of the Arm, Shoulder and Hand

	NOT AT ALL	SLIGHTLY	MODERATELY	QUITE A BIT	EXTREMELY
22. During the past week, *to what extent* has your arm, shoulder or hand problem interfered with your normal social activities with family, friends, neighbours or groups? *(circle number)*	1	2	3	4	5

	NOT LIMITED AT ALL	SLIGHTLY LIMITED	MODERATELY LIMITED	VERY LIMITED	UNABLE
23. During the past week, were you limited in your work or other regular daily activities as a result of your arm, shoulder or hand problem? *(circle number)*	1	2	3	4	5

Please rate the severity of the following symptoms in the last week. *(circle number)*

	NONE	MILD	MODERATE	SEVERE	EXTREME
24. Arm, shoulder or hand pain.	1	2	3	4	5
25. Arm, shoulder or hand pain when you performed any specific activity.	1	2	3	4	5
26. Tingling (pins and needles) in your arm, shoulder or hand.	1	2	3	4	5
27. Weakness in your arm, shoulder or hand.	1	2	3	4	5
28. Stiffness in your arm, shoulder or hand.	1	2	3	4	5

	NO DIFFICULTY	MILD DIFFICULTY	MODERATE DIFFICULTY	SEVERE DIFFICULTY	SO MUCH DIFFICULTY THAT I CAN'T SLEEP
29. During the past week, how much difficulty have you had sleeping because of the pain in your arm, shoulder or hand? *(circle number)*	1	2	3	4	5

	STRONGLY DISAGREE	DISAGREE	NEITHER AGREE NOR DISAGREE	AGREE	STRONGLY AGREE
30. I feel less capable, less confident or less useful because of my arm, shoulder or hand problem. *(circle number)*	1	2	3	4	5

DASH DISABILITY/SYMPTOM SCORE = $\left[\left(\frac{\text{sum of n responses}}{n}\right) - 1\right] \times 25$, where n is equal to the number of completed responses.

A DASH score may not be calculated if there are greater than 3 missing items.
